# An interlaboratory comparison of ITS2-PCR for the identification of yeasts, using the ABI Prism 310 and CEQ8000 capillary electrophoresis systems

**DOI:** 10.1186/1471-2180-5-14

**Published:** 2005-03-18

**Authors:** Thierry De Baere, Anne Van Keerberghen, Peter Van Hauwe, Hans De Beenhouwer, An Boel, Gerda Verschraegen, Geert Claeys, Mario Vaneechoutte

**Affiliations:** 1 Department of Clinical Chemistry, Microbiology and Immunology, University Hospital Ghent, Belgium; 2 Laboratory Microbiology, Onze Lieve Vrouw Ziekenhuis, Aalst, Belgium

## Abstract

**Background:**

Currently, most laboratories identify yeasts routinely on the basis of morphology and biochemical reactivity. This approach has quite often limited discriminatory power and may require long incubation periods. Due to the increase of fungal infections and due to specific antifungal resistence patterns for different species, accurate and rapid identification has become more important. Several molecular techniques have been described for fast and reliable identification of yeast isolates, but interlaboratory exchangeability of identification schemes of molecular techniques has hardly been studied. Here, we compared amplified ITS2 fragment length determination by an ABI Prism 310 (Applied Biosystems, Foster City, Ca.) capillary electrophoresis system with that obtained by a CEQ8000 (Beckman Coulter, Fullerton, Ca.) capillary electrophoresis system.

**Results:**

Although ITS2 size estimations on both systems differed and separate libraries had to be constructed for each system, both approaches had the same discriminatory power with regard to the 44 reference strains, identical identifications were obtained for 39/ 40 clinical isolates in both laboratories and strains from 51 samples were correctly identified using CEQ8000, when compared to phenotypic identification.

**Conclusion:**

Identification of yeasts with ITS2-PCR followed by fragment analysis can be carried out on different capillary electrophoresis systems with comparable discriminatory power.

## Background

The clinical importance of yeast infections has increased during the last decades, not only because the number of yeast infections has increased, but also because yeast infections have become a frequent cause of morbidity and mortality in immunocompromised patients [1]. Besides the higher frequency there is also an important change in the spectrum of the species causing clinical infections. Whereas *Candida albicans *has long been considered as the clinically most important species of the genus, the occurrence and pathogenic importance of the non-albicans species has been increasing steadily [2]. Because antifungal susceptibility is differing between the species, rapid and reliable identification of the clinical isolates can contribute to an efficient therapy of the patient [3]. The conventional identification of yeast isolates depends on biochemical properties such as assimilation and fermentation reactions and morphology [4], features which are not always stable, which are often not easy to interpret, and which may be time-consuming [5]. Biochemical identification has been standardized and (semi-)automated (e.g. Vitek-2) [6], but this approach equally suffers from the limitations of phenotypic identification in general. As a solution for those problems, several PCR-based methods have been described. Some of these approaches rely on species-specific probes [7,8] – being limited to those species for which probes are available, but most of them are based on amplification using universal fungal primers, followed by post-amplification analysis like probe hybridization [9], sequencing [10,11], restriction analysis [12,13], temperature gradient gel electrophoresis [14] or – most simply and least laborious – fragment length determination [15-18]. In addition, fingerprinting techniques primarily developed for strain typing are also used for identification: RAPD [19] and AFLP [20,21]. Finally, techniques like real-time PCR [22,23], pyrosequencing [24] and Luminex flow cytometry [25] have been used for identification of yeasts.

The principle of Internal Transcribed Spacer 2 (ITS2)-PCR was initially described by Turenne *et al. *[13]. Amplification of the ITS2-PCR is followed by capillary electrophoresis for the precise determination of the fragment length, whereby the length of the fragment is used for identification. Evaluation, adaptation and expansion of the number of species included has been carried out at our laboratory and has resulted in a publicly available database, listing the ITS2-sizes for 39 yeast species [16]. Subsequently, an interlaboratory evaluation indicated that the ITS2-PCR technique and the database could be exchanged between different laboratories, when using the same electrophoresis platform, i.e. ABI310 (Applied Biosystems, Foster City, Ca.) [17].

Our present aim was to evaluate the interlaboratory exchangeability of the technique between laboratories using different capillary electrophoresis systems, i.e. ABI310 versus CEQ8000 (Beckman Coulter, Fullerton, Ca.), for the size determination of the amplified ITS2-fragments.

## Results

### Construction of an ITS2-length library using CEQ8000

Since ITS2-fragment length determination on CEQ8000 was expected to be different from that obtained on ABI310, we determined the length of a set of 44 reference strains, representing 33 species and subspecies, using the CEQ8000 capillary electrophoresis apparatus. The obtained lengths on CEQ8000 are listed in Table 1 (Additional file 1) and are compared with the lengths obtained on ABI310, as reported earlier [16], and with the theoretically expected lengths, as derived from published sequences.

It could be observed that the values obtained on the CEQ8000 were always higher than on ABI310. The size differences were in the range of 0.3 basepairs (bp) for *C. neoformans *subsp. *gattii *(strain IHEM 04170) to 7.2 bp for *Malassezia furfur *(strain IHEM 03967), with an average of 2.61 bp (SD 1.31 bp). There was no correlation between fragment size and size difference and there was no constant size difference. As illustrated in Table 1 (Aditional file 1), we could neither establish a correlation between %GC-content of the ITS2-region and migration differences as observed on both machines.

The accuracy of fragment length calculation by CEQ8000 seemed to be somewhat higher than that achieved by ABI310, since in 33 of 44 cases the calculated length on CEQ8000 was closer to the theoretical length than that calculated on ABI310 (Table 1, Additional file 1). Although the absolute lengths obtained on CEQ8000 differed from those on ABI310, fragment length determination of the amplified ITS2 region on CEQ8000 enabled discrimination between the same species that could be differentiated on the ABI310. All species included could be identified to species level, except for the members of the genus *Trichosporon*, for which four of the five tested species had the same ITS2-fragment length. Since none of all other yeast species tested had an ITS2 of this length, the *Trichosporon *species could be identified as a group.

### Applicability

The obtained ITS2-fragment lengths for the different species on CEQ8000 (Table 1, Additional file 1) were used as reference values with which ITS2-lengths of unknowns were compared.

ITS2-fragment length determination on the CEQ8000 enabled the identification of the yeasts present in the cultures of 51 clinical samples, of which 5 samples contained different yeast species simultaneously. The following species and mixtures of species were identified: *C. albicans *(n = 12), *C. albicans *+ *C. glabrata *(1), *C. albicans *+ *C. parapsilosis *(1), *C. glabrata *(11), *C. guilliermondii *(1), *C. kefyr *(1), *C. parapsilosis *(12), *C. tropicalis *(7), *C. tropicalis *+ *C. albicans *(1), *C. tropicalis *+ *C. albicans *+ *C. glabrata *(1), *C. tropicalis *+ *C. glabrata *(1), *C. krusei *(1), and *Ustilago maydis *(1). No misidentifications occurred, compared to phenotypic identification.

### Exchangeability of data

To evaluate the exchangeability of the technique between two laboratories using a different capillary electrophoresis apparatus, clinical strains were exchanged. Laboratory G identified 20 clinical isolates from the Ghent University Hospital on ABI310 and sent these to laboratory A, where identification was carried out on CEQ8000. Laboratory A identified the clinical isolates from 20 cultured samples from the Onze Lieve Vrouw Ziekenhuis Aalst, using the CEQ8000 and sent these to laboratory G, where identification was carried out on ABI310. The results of this comparison are presented in Table 2 (Additional file 2). The identification obtained with ITS2-PCR in both laboratories was in agreement for 39 of the 40 cultures. One culture yielded another identification, respectively as *C. albicans *using the CEQ8000 and as *C. krusei *by means of the ABI310. Retesting at laboratory A indicated that the sample contained a mixture of both species, while repeated culture and retesting at laboratory G yielded again only *C. krusei*.

Based on culture results, one sample (OLVA 5397) was thought to contain a mixture of *C. albicans *and a non-albicans isolate. Both ITS2-PCR identification systems revealed that the mixture contained in fact three species, namely *C. albicans*, *C. tropicalis *and *C. glabrata*.

During this study, one isolate was misidentified in both laboratories using ITS2-PCR. An isolate identified biochemically as *C. dubliniensis*, was identified as *C. krusei *by means of ITS2-PCR in both laboratories. ITS2-sequencing, whereby the obtained sequence was compared to all known sequences in Genbank using BLAST, confirmed the biochemical identification as *C. dubliniensis*. The ITS2-sequence showed highest sequence similarity to *C. dubliniensis*, but contained a 2-bp indel (insertion/deletion) compared to the ITS2 sequence of the *C. dubliniensis *reference strains that had been used to construct the ITS2-length library. Genbank contains 17 ITS2 sequences of *C. dubliniensis*, of which 6 have the 2-bp indel.

The ITS2 size library lists *C. krusei *as having a two bp shorter ITS2 size than that of *C. dubliniensis *and this explains the misidentification. This finding was added to the library, cautioning that in some cases *C. dubliniensis *strains with an aberrant ITS2 fragment length may occur and thus may result in a misidentification as *C. krusei*.

## Discussion and conclusions

ITS2-PCR followed by fragment length determination by capillary electrophoresis on ABI310 (Applied Biosystems) has been shown to be a fast and efficient identification technique for clinically important yeast species [16,18] and for which data are exchangeable between laboratories using the same capillary electrophoresis system [17]. Here we evaluated whether comparative discriminatory power could be obtained on another capillary electrophoresis apparatus, *in casu *on CEQ8000, and whether identifications obtained in different laboratories using different capillary electrophoresis systems were identical.

Since the fragment length estimation differs between different capillary electrophoresis systems, it was found necessary to recreate an ITS2 length library for the CEQ8000. The different size estimation most probably results from migration differences between different systems, caused by the usage of different fluorescent labels on the primers and of different polymers in the capillaries. Also the use of different marker fragments may result in differences between calculated lengths. It was observed that size estimations on CEQ8000 were always higher than on ABI310, ranging from 0.3 to 7.2 bp differences, without any notable consistency in the observed differences.

However, differences were constant for each species, such that a new library for CEQ8000 could be constructed. It was found that the species that could be differentiated from each other by ITS2 length determination on ABI310 could also be differentiated by using the CEQ8000.

ITS2-PCR was not very useful for differentiation between species of the genus *Trichosporon*. Identification of *Trichosporon *species was also impossible by ITS2-PCR on the ABI310 [16], and even sequencing of the ITS2 region is not always sufficient for final discrimination between all the species [26]. A more reliable identification of *Trichosporon *spp. can be obtained by sequencing a more variable region like IGS1 (intergenetic spacer region 1) [27] or making use of species specific probes and detection of hybridization in the Luminex flow cytometer [25].

During this study, both an additional problem and an additional possiblity of ITS2 fragment length determination for the identification of yeast species was encountered.

Apparently, strains with aberrant ITS2 lengths occur. Here, a strain of which the biochemical identification as *C. dubliniensis *was confirmed by ITS2-sequence determination was misidentified by both laboratories as *C. krusei*, when identification was based on ITS-length determination. It could be shown that this was the result of a 2 bp indel of the ITS2-region of this strain, an indel which is present in 6 of the 17 published *C. dubliniensis *sequences. Further research is needed to find out whether the subdivision that can be made on the basis of ITS2-length corresponds to epidemiological or clinical relevance. An additional advantage of ITS2-PCR was observed when a mixed culture was studied. In order to identify the separate species present in mixed cultures, isolation of the cultures is necessary when identification is based on biochemical features or on sequencing of genes. However, when using ITS2-PCR on either CEQ8000 or ABI310, the different species present can be identified immediately, since one will obtain an ITS2-fingerprint composed of several peaks, each with a length corresponding to the respective species present in the mixed sample.

Our results obtained on mixed culture samples indicate that ITS2-PCR could be applied for direct, non culture based, simultaneous detection of different species from a clinical sample. Whether this will be possible, will only depend on the strength of the DNA-extraction method used.

## Methods

### Yeast strains

A set of 44 reference strains or well-documented strains belonging to 33 species and subspecies, which were reference and type strains from culture collections or for which the correct identification had been obtained by sequencing of the ITS2 region in previous studies [16,17], were used as reference panel (Table 1, Additional file 1).

A series of 40 cultures on Sabouraud agar (Becton Dickinson, Erembodegem, Belgium) – 20 from each hospital – of clinical samples with unknown yeast isolates was also studied. Routine identification of these isolates was carried out using Auxacolor (Sanofi-Pasteur, Marnes-la-Coquette, France) at the Ghent University Hospital (GUH) laboratory (Laboratory G) and using Chromagar (Novolab, Geraardsbergen, Belgium) and API 32C (bioMérieux, Marcy-l'Etoile, France) at the Onze Lieve Vrouw Ziekenhuis Aalst (OLVA) laboratory (Laboratory A).

The selection of 40 cultures contained 43 clinical isolates belonging to the following species: *C. albicans *(n = 9), *C. dubliniensis *(1), *C. glabrata *(5), *C. guilliermondii *(3), *C. kefyr *(2), *C. lusitaniae *(1), *C. parapsilosis *(8), *C. rugosa *(1), *C. tropicalis *(6), *Debaryomyces hansenii *(1), *C. krusei *(5) and *Ustilago maydis *(1). All 40 culture plates were exchanged between laboratories, to study whether the identifications obtained in one laboratory could be confirmed in the other.

### DNA-extraction, PCR, capillary electrophoresis and analysis

#### a. DNA-extraction

##### Laboratory A

DNA was extracted by alkaline lysis. A loopful (1μl inoculation needle) of yeast colonies was suspended in 40 μl of 0.05 M NaOH / 0.25 M SDS and incubated for 15 minutes at 95°C, whereafter 500 μl milliQ water was added.

##### Laboratory G

DNA-extraction was carried out using a boilling-freezing method. A loopful of yeast colonies was suspended in 250 μl TE-buffer, heated for 15 minutes at 95°C and immediately frozen at -70°C for at least 15 minutes.

#### b. PCR

##### Laboratory A

PCR reactions were carried out in 50 μl reactions containing 10 μl yeast DNA extract, 1 x PCR buffer (Invitrogen, Carlsbad, Ca.), 0.2 mM of each dNTP, 1.5 mM MgCl_2_, 5 U of *Taq *polymerase (Invitrogen) and 0.5 μM of primers ITS4 TCCTCCGCTTATTGATATGC) and ITS86 (GTGAATCATCGAATCTTTGAAC). Primer ITS86 was labeled with Beckman dye D4 (Proligo, Boulder, Co.). The amplification was carried out with the following protocol: 5 min at 94°C, 30 cycli of 1 min at 94°C, 1 min at 55°C and 1 min at 72°C and a final extension of 7 min at 72°C, followed by cooling at 4°C.

##### Laboratory G

PCR reactions were carried out in 25 μl reactions containing 2.5 μl yeast DNA extract, PCR Master Mix (Qiagen, Hilden, Germany) and 0.5 μM of primers ITS4 and ITS86 (sequence see above). Primer ITS86 was HEX-labeled (Applied Biosystems). The amplification was carried out with the following protocol: 5 min at 94°C, 30 cycli of 1 min at 94°C, 1 min at 55°C and 1 min at 72°C and a final extension of 7 min at 72°C, followed by cooling at 4°C.

#### c. Capillary electrophoresis

##### Laboratory A

Capillary electrophoresis was performed on the eight capillary system CEQ8000. PCR products were diluted tenfold in nuclease-free water (Sigma, St. Louis, Mo.). Four μl of the PCR dilution was combined with 40 μl of SLS loading mix (deionized formamide, Analis, Namur, Belgium) and 0.5 μl of the CEQ DNA Size standard kit-600 (Analis). Samples were run on the CEQ8000 under the standard method FRAG-4: capillary temperature of 50°C, denaturing temperature of 90°C for 120 seconds, injection voltage of 2.0 kV for 30 seconds and separation voltage of 4.8 kV for 60 minutes. The total running time for one row of eight samples was 85 minutes.

##### Laboratory G

The capillary electrophoresis apparatus used was the ABI Prism 310 Genetic Analyser (Applied Biosystems, Foster City, Ca.). Electrophoresis was done in a single capillary, filled with liquid polymer (POP-4: Performance Optimized Polymer, Applied Biosystems). One μl of the PCR-product was added to the electrophoresis mixture (0.1 μl HD 400 marker, 0.3 μl of the ROX-500 marker (both from Applied Biosystem) and 12.1 μl of deionized formamide. All samples were first denaturated (2 min 95°C) before placement on the capillary. Injection of the sample was carried out at 5 kV for 10 sec, followed by an electrophoreis at 60°C and 15 kV, during 35 min.

#### d. Size determination of the fragment length, followed by identification

At laboratory A, the size and identity of the amplified ITS2-fragment was determined by the Fragment Analysis Module of the CEQ8000. The specific parameters concerning the marker standard used and the reference library, containing the ITS2 fragment lengths of the reference strains needed for identification, have to be selected by the user. The program then automatically calculates the size of the amplified ITS2-fragment by means of the known lengths of the fragments of the CEQ DNA Size standard kit-600 (Analis). This calculated fragment length is then compared to the ITS2 fragment lengths present in the selected library and as a result, the Fragment Analysis Module of the CEQ8000 gives an identification of the unknown strain.

At laboratory G, the derivation of the fragment length is carried out by the Gene Scan analysis software (Applied Biosystems). The results were presented in a table indicating length and intensity of the observed fragments. The in-house software BaseHopper [16] enables to compare the obtained fragment length to those lengths present in the ITS2-database.

## Authors' contributions

GC, GV, AB and HD were responsible for sample collection and initial biochemical identification. TDB, PVH and AV carried out moleculair biological identification, analysis of the data. TDB, AV and MV drafted the manuscript. All authors read and approved the final manuscript.

## Supplementary Material

Additional File 1 Table 1. List of reference strains used, with the ITS2 fragment lengths obtained on CEQ8000 and ABI310 capillaries, and theoretical fragment lengths as calculated using Genbank seqeunces. ^a^: ATCC: American Type Culture Collection, Rockville, Md.; AZB: Algemeen Ziekenhuis Jette Brussel, Belgium; DGG: Veterinary Medicine (Diergeneeskunde), University Ghent, Belgium; HHR: Heilig Hartziekenhuis, Roeselare, Belgium; IHEM: Institute Hygiene Epidemiology Mycology; GUH : Ghent University Hospital, Belgium. ^b^: CEQ-TL: Difference between ITS2-length as determined on CEQ8000 with that as calculated from GenBank sequences (TL: theoretical length); ABI-TL: difference between ITS2-length as determined on ABI310 with that as calculated from GenBank sequences. Click here for file

Additional File 2 Table 2. Comparison of the identifications obtained independently on ABI310 and CEQ8000 in the two different laboratories. ^a^: Confirmed: the identification was first obtained in the other laboratory and confirmed by this laboratory. ^b^: Results of two independent tests are separated by slash. Click here for file
