# Supplementary material for: An interlaboratory comparison of ITS2-PCR for the identification of yeasts, using the ABI Prism 310 and CEQ8000 capillary electrophoresis systems
Source: BMC Microbiol. 2005 Mar 18;5:14. doi: 10.1186/1471-2180-5-14 (PMC1082908; doi:10.1186/1471-2180-5-14)
Supplement: Additional File 1 — Table 1. List of reference strains used, with the ITS2 fragment lengths obtained on CEQ8000 and ABI310 capillaries, and theoretical fragment lengths as calculated using Genbank seqeunces. a: ATCC: American Type Culture Collection, Rockville, Md.; AZB: Algemeen Ziekenhuis Jette Brussel, Belgium; DGG: Veterinary Medicine (Diergeneeskunde), University Ghent, Belgium; HHR: Heilig Hartziekenhuis, Roeselare, Belgium; IHEM: Institute Hygiene Epidemiology Mycology; GUH : Ghent University Hospital, Belgium. b: CEQ-TL: Difference between ITS2-length as determined on CEQ8000 with that as calculated from GenBank sequences (TL: theoretical length); ABI-TL: difference between ITS2-length as determined on ABI310 with that as calculated from GenBank sequences. [file 1471-2180-5-14-S1.pdf]

Species	Original Number	ITS2-Length (bp)		Difference (bp)	Theoretical length (bp)	CEQ-TL	ABI-TL	GC%	Genbank accession used
		on CEQ8000	on ABI310						
Arxiozyma telluris	DGG 05036	374.5	371.9	2.6	374	0.5	-2.1	52.3	AJ871396
Blastoschizomyces capitatum	IHEM 05091	253.3	250.7	2.6	251	2.3	-0.3	34.8	AF455443
B. capitatum	HHR 0303 14240	254.8	251.1	3.7	251	3.8	0.1	34.8	AF455443
Candida albicans	IHEM 03731	284.6	281.6	3.0	284	0.6	-2.4	48.2	AF217609
C. albicans	IHEM 03243	284.5	281.4	3.1	284	0.5	-2.6	48.2	AF217609
C. dubliniensis	IHEM 14280	288.7	286.2	2.5	289	-0.3	-2.8	47.1	AF218993
C. glabrata	IHEM 04566	364.0	362.4	1.6	365	-1.0	-2.6	46.9	AF218994
C. glabrata	IHEM 04210	364.1	362.4	1.7	365	-0.9	-2.6	46.9	AF218994
C. guilliermondii	IHEM 01067	326.5	324.1	2.4	325	1.5	-0.9	45.9	AF218996
C. guilliermondii	IHEM 01879	327.1	323.1	3.9	325	2.1	-1.9	45.9	AF218996
C. kefyr	IHEM 04211	376.5	375.5	1.0	378	-1.5	-2.5	47.1	AF218997
C. kefyr	IHEM 04592	376.4	375.5	0.9	378	-1.6	-2.5	47.1	AF218997
C. krusei	ATCC 6258	287.1	285.3	1.8	294	-6.9	-8.7	56.5	L47113
C. krusei	IHEM 04562	287.2	285.2	2.0	294	-6.8	-8.8	56.5	L47113
C. lipolytica	IHEM 01958	190.2	188.1	2.1	190	0.2	-1.9	44.2	AF218983
C. lusitaniae	IHEM 04593	202.9	200.3	2.5	205	-2.2	-4.7	51.7	AF218970
C. lusitaniae	IHEM 10293	202.6	200.2	2.4	205	-2.4	-4.8	51.7	AF218970
C. parapsilosis	IHEM 02305	256.1	253.8	2.3	262	-5.9	-8.2	43.9	AY391844
C. parapsilosis	IHEM 02052	255.8	253.7	2.1	262	-6.2	-8.3	43.9	AY391844
C. rugosa	GUH 0302 93803	216.0	214.8	1.2	223	-7.0	-8.2	49.3	AF218971
C. sake	IHEM 17939	235.7	233.0	2.7	237	-1.3	-4.0	42.2	AJ549822
C. tropicalis	IHEM 05609	273.4	270.2	3.2	272, 273, 274			41.6-41.9	AF219000, AF219001, AF218992
C. tropicalis	IHEM 10285	274.2	271.4	2.8	272, 273, 274			41.6-41.9	AF219000, AF219001, AF218992
Cryptococcus albidus	IHEM 03267	354.9	352.7	2.2	353	1.9	-0.3	45.3	AF218972
C. humicola	AZB 98-043	299.0	295.8	3.2	300	-1.0	-4.2	48.0	AF201298
C. laurentii	GUH 0203 93854	309.9	307.9	2.0	310	-0.1	-2.1	50.3	AJ421006
C. neoformans subsp. *neoformans*	IHEM 04171	320.9	317.9	3.0	320	0.9	-2.1	48.4	AF218975
C. neoformans subsp. *neoformans*	IHEM 04165	320.5	318.4	2.1	320	0.5	-1.6	48.4	AF218975
C. neoformans subsp. *gattii*	IHEM 04170	320.3	320.0	0.3	321	-0.7	-1.0	47.4	AF196312
Debaryomyces hansenii	HHR 0301 10394	329.0	325.2	3.8	327	2.0	-1.8	41.3	AF218984
Dekkera bruxellensis	GUH 0111 93801	264.3	261.8	2.5	264	0.3	-2.2	44.7	AJ871395
Filobasidium uniguttulatum	GUH 0302 93802	381.8	379.4	2.4	378	3.8	1.4	43.4	AF218985
Geotrichum candidum	IHEM 06284	195.9	193.4	2.5	195	0.9	-1.6	38.5	AF157596
Malassezia furfur	IHEM 03967	501.6	494.4	7.2	501	0.6	-6.6	54.3	AB105151
M. pachydermatis	DGG 04541	461.7	455.6	6.1	461	0.7	-5.4	51.4	AF246896
Pichia norvegensis	GUH 0211 93821	267.8	267.1	0.7	272	-4.2	-4.9	55.5	AB118938
Saccharomyces cerevisiae	IHEM 14402	369.2	366.2	3.0	365, 366, 367			42.2-42.5	AF219005, AF219006, AF219007
S. cerevisiae	IHEM 14542	369.4	366.3	3.1	365, 366, 367			42.2-42.5	AF219005, AF219006, AF219008
Trichosporon asahii	IHEM 17910	302.1	301.4	0.7	304	-1.9	-2.6	45.1	AF245218
T. asahii	IHEM 09334	302.8	300.6	2.2	304	-1.2	-3.4	45.1	AF245218
T. asteroides	IHEM 10214	303.3	298.6	4.7	304	-0.7	-5.4	45.4	AF444416
T. inkin	IHEM 05824	303.2	298.7	4.5	304	-0.8	-5.3	46.1	AF444420
T. mucoides	IHEM 13920	298.9	297.1	1.8	297	1.9	0.1	48.8	AF444423
T. ovoides	IHEM 09336	302.0	296.4	2.5	303	-1.0	-6.6	44.9	AF444439
Average (bp)				2.61		-0.8	-3.4		
Standard deviation (bp)				1.31		2.75	2.63		
