# Supplementary material for: An interlaboratory comparison of ITS2-PCR for the identification of yeasts, using the ABI Prism 310 and CEQ8000 capillary electrophoresis systems
Source: BMC Microbiol. 2005 Mar 18;5:14. doi: 10.1186/1471-2180-5-14 (PMC1082908; doi:10.1186/1471-2180-5-14)
Supplement: Additional File 2 — Table 2. Comparison of the identifications obtained independently on ABI310 and CEQ8000 in the two different laboratories. a: Confirmed: the identification was first obtained in the other laboratory and confirmed by this laboratory. b: Results of two independent tests are separated by slash. [file 1471-2180-5-14-S2.pdf]

**Sample number**	**Identification CEQ8000 (Laboratory A)**	**Identification ABI310 (Laboratory G)**
OLVA 3961	*Candida albicans*	Confirmed
OLVA 4091	*C. albicans*	Confirmed
OLVA 4203	*C. albicans*	Confirmed
OLVA 6006	*C. albicans*	Confirmed
OLVA 5397	*C. albicans + C. tropicalis + C. glabrata*	Confirmed
OLV A 4863	*C. glabrata*	Confirmed
OLVA 5408	*C. glabrata*	Confirmed
OLVA 6007	*C. glabrata*	Confirmed
OLVA 4B2	*C. guilliermondii*	Confirmed
OLVA 4B7	*C. lusitaniae*	Confirmed
OLVA 4180	*C. parapsilosis*	Confirmed
OLVA 4542	*C. parapsilosis*	Confirmed
OLVA 4862	*C. parapsilosis*	Confirmed
OLVA 5738	*C. parapsilosis*	Confirmed
OLVA 4B3	*C. parapsilosis*	Confirmed
OLVA 5008	*C. tropicalis*	Confirmed
OLVA 5409	*C. tropicalis*	Confirmed
OLVA 4B6	*C. tropicalis*	Confirmed
OLVA 3462	*C. krusei*	Confirmed[a]
OLVA 3859	*Ustilago maydis*	Confirmed
GUH 0312 808	Confirmed	*Candida albicans*
GUH 0401 808	Confirmed	*C. albicans*
GUH 0401 824	Confirmed	*C. albicans*
GUH 0401 826	Confirmed	*C. dubliniensis*
GUH 0306 805	Confirmed	*C. glabrata*
GUH 0307 830	Confirmed	*C. guilliermondii*
GUH 0309 800	Confirmed	*C. guilliermondii*
GUH 0307 834	Confirmed	*C. kefyr*
GUH 0309 802	Confirmed	*C. kefyr*
GUH 0310 805	Confirmed	*C. parapsilosis*
GUH 0311 807	Confirmed	*C. parapsilosis*
GUH 0401 818	Confirmed	*C. parapsilosis*
GUH 0310 804	Confirmed	*C. rugosa*
GUH 0308 819r	Confirmed	*C. tropicalis*
GUH 0401 825	Confirmed	*C. tropicalis*
GUH 0307 933	Confirmed	*Debaryomyces hansenii*
GUH 0307 831	Confirmed	*C. krusei*
GUH 0308 819w	Confirmed	*C. krusei*
GUH 0309 833	Confirmed	*C. krusei*
GUH 0307 829[b]	*Candida albicans* / *C. albicans + C. krusei*	*C. krusei / C. krusei*
